# Supplementary material for: Decoding the human brain during intelligence testing
Source: Commun Biol. 2025 Dec 23;9:90. doi: 10.1038/s42003-025-09354-4 (PMC12820092; doi:10.1038/s42003-025-09354-4)
Supplement: Supplementary file 2 — Reporting Summary [file 42003_2025_9354_MOESM2_ESM.pdf]

nature portfolio

Corresponding author(s): Kirsten Hilger

Last updated by author(s): Nov 16, 2025

Reporting Summary

Nature Portfolio wishes to improve the reproducibility of the work that we publish. This form provides structure for consistency and transparency in reporting. For further information on Nature Portfolio policies, see our Editorial Policies and the Editorial Policy Checklist.

Statistics

For all statistical analyses, confirm that the following items are present in the figure legend, table legend, main text, or Methods section.

n/a	Confirmed	
☐	☒	The exact sample size (*n*) for each experimental group/condition, given as a discrete number and unit of measurement
☐	☒	A statement on whether measurements were taken from distinct samples or whether the same sample was measured repeatedly
☐	☒	The statistical test(s) used AND whether they are one- or two-sided<br>*Only common tests should be described solely by name; describe more complex techniques in the Methods section.*
☐	☒	A description of all covariates tested
☐	☒	A description of any assumptions or corrections, such as tests of normality and adjustment for multiple comparisons
☐	☒	A full description of the statistical parameters including central tendency (e.g. means) or other basic estimates (e.g. regression coefficient) AND variation (e.g. standard deviation) or associated estimates of uncertainty (e.g. confidence intervals)
☐	☒	For null hypothesis testing, the test statistic (e.g. *F*, *t*, *r*) with confidence intervals, effect sizes, degrees of freedom and *P* value noted<br>*Give P values as exact values whenever suitable.*
☒	☐	For Bayesian analysis, information on the choice of priors and Markov chain Monte Carlo settings
☒	☐	For hierarchical and complex designs, identification of the appropriate level for tests and full reporting of outcomes
☐	☒	Estimates of effect sizes (e.g. Cohen's *d*, Pearson's *r*), indicating how they were calculated

Our web collection on statistics for biologists contains articles on many of the points above.

Software and code

Policy information about availability of computer code

Data collection	We reused datasets from two independent laboratories. The first sample included data previously published by Vakhtin et al. (2014): https://doi.org/10.1016/j.neuroimage.2014.09.055. These data were recorded under supervision of Rex Jung (University of New Mexico) at the Mind Research Network (MRN), Albuquerque, NM. The included fMRI data were recorded on a 3-T Siemens Trio scanner. The second dataset was provided by the research group of Adam Chuderski (Centre for Cognitive Science, Jagiellonian University in Krakow), including data published by Ociepka et al. (2023): https://doi.org/10.1016/j.intell.2023.101780. EEG data were recorded using a Biosemi ActiveTwo system.
Data analysis	Data and analyses code have been made freely available. EEG data: https://osf.io/kv2sx (resting state), https://osf.io/htrsg (RPM). Code for data analysis and fMRI data (preprocessed) are available on GitHub and Zenodo: https://github.com/jonasAthiele/connectors_intelligence https://doi.org/10.5281/zenodo.17623340

For manuscripts utilizing custom algorithms or software that are central to the research but not yet described in published literature, software must be made available to editors and reviewers. We strongly encourage code deposition in a community repository (e.g. GitHub). See the Nature Portfolio guidelines for submitting code & software for further information.

Data

Policy information about availability of data

All manuscripts must include a data availability statement. This statement should provide the following information, where applicable:

- Accession codes, unique identifiers, or web links for publicly available datasets
- A description of any restrictions on data availability
- For clinical datasets or third party data, please ensure that the statement adheres to our policy

Data and analyses code have been made freely available. EEG data: https://osf.io/kv2sx (resting state), https://osf.io/htrsg (RPM). Code for data analysis and fMRI data (preprocessed) are available on GitHub and Zenodo: https://github.com/jonasAthiele/connectors_intelligence https://doi.org/10.5281/zenodo.17623340

Research involving human participants, their data, or biological material

Policy information about studies with human participants or human data. See also policy information about sex, gender (identity/presentation), and sexual orientation and race, ethnicity and racism.

Reporting on sex and gender	Information on sex was provided in both datasets and is reported in the population characteristics. It was used as a control variable.
Reporting on race, ethnicity, or other socially relevant groupings	No socially constructed or socially relevant categorization variables were used in this research. All analyses were controlled for age, sex (EEG and fMRI analyses), mean frame-wise displacement (fMRI analysis), and for the number of removed epochs due to artifacts (EEG analysis).
Population characteristics	Sample 1: Consisted of 67 participants (26 females; age range: 18-29 years, mean age: 22.91 years, age SD: 3.19 years; 56 right-handed, 9 left-handed, 2 with missing handedness data). Participants were screened and excluded if they reported past major head injuries, psychiatric or neurological disorders, substance abuse, or consumption of any psychoactive medications.<br><br>Sample 2: Consisted of 131 participants (65 females, age range: 18-40 years, M: 23.63, SD: 4.31, all right-handed). All participants had normal or corrected-to-normal vision, and no history of neurological problems.
Recruitment	Sample 1: Participants were recruited from the University of New Mexico (UNM), Albuquerque, NM, USA. See: Vakhtin et al. (2014): https://doi.org/10.1016/j.neuroimage.2014.09.055.<br><br>Sample 2: Adult participants were recruited via internet advertisements from a general population of a large academic city in Central Europe. See: Ociepka et al. (2023): https://doi.org/10.1016/j.intell.2023.101780
Ethics oversight	All ethical regulations relevant to human research participants were followed.<br><br>Sample 1: Procedures were approved by the UNM Institutional Review Board, and all procedures were implemented in accordance with the Declaration of Helsinki.<br><br>Sample 2: Procedures were approved by the local ethics board, informed consent was obtained from each participant, and all procedures were implemented in accordance with the Declaration of Helsinki.

Note that full information on the approval of the study protocol must also be provided in the manuscript.

Field-specific reporting

Please select the one below that is the best fit for your research. If you are not sure, read the appropriate sections before making your selection.

☐ Life sciences ☒ Behavioural & social sciences ☐ Ecological, evolutionary & environmental sciences

For a reference copy of the document with all sections, see nature.com/documents/nr-reporting-summary-flat.pdf

Behavioural & social sciences study design

All studies must disclose on these points even when the disclosure is negative.

Study description	The study is a quantitative cross-sectional design. For each participant fMRI or EEG data were recorded during the performance of an intelligence test. Graph-theoretical measures (participation coefficient and degree) of functional brain connectivity (fMRI) and multiscale entropy (EEG) were extracted and correlated with individual intelligence test performance (Raven Progressive Matrices scores).
Research sample	We reused datasets from two independent laboratories.<br><br>Sample 1 (Vakhtin et al. 2014: https://doi.org/10.1016/j.neuroimage.2014.09.055): The final sample consisted of 67 healthy

	participants (26 females; age range: 18-29 years, age mean (M): 22.91 years, age standard deviation (SD): 3.19 years; 56 right-handed, 9 left-handed, 2 with missing handedness data).<br><br>Sample 2 (Ociepka et al. (2023): https://doi.org/10.1016/j.intell.2023.101780): The final sample included 131 healthy participants (65 females, age range: 18-40 years, age M: 23.63, age SD: 4.31, all right-handed).
Sampling strategy	Since we reused datasets, details about the sampling strategy are provided elsewhere. Specifically, details on the first dataset are provided in Vakhtin et al. (2014): https://doi.org/10.1016/j.neuroimage.2014.09.055; details on the second dataset are provided in Ociepka et al. (2023): https://doi.org/10.1016/j.intell.2023.101780.
Data collection	Details on data collection can be found in the references provided above.
Timing	Details on timing can be found in the references provided above.
Data exclusions	Sample 1: 85 healthy participants (34 females, age range: 18-29 years, age M: 22.35; age SD: 3.14) that completed all RPM runs were provided by Vakhtin et al. 2014 https://doi.org/10.1016/j.neuroimage.2014.09.055. After exclusions due to excessive head motion and short data length (see section Functional MRI Preprocessing and section Statistics and Reproducibility), the final sample consisted of 67 participants (26 females; age range: 18-29 years, age M: 22.91 years, age SD: 3.19 years; 56 right-handed, 9 left-handed, 2 with missing handedness data).<br><br>Sample 2: 161 right-handed participants were provided by Ociepka et al. (2023): https://doi.org/10.1016/j.intell.2023.101780 (77 females, age range: 18-40 years, M: 23.60, SD: 4.28). Participants were excluded if demographic information (age or sex) was missing, as these were used as control variables. Additionally, participants with an insufficient number of usable trials due to artifact rejection were excluded (see section Statistics and Reproducibility for details). This resulted in 131 participants (65 females, age range: 18-40 years, age M: 23.63, age SD: 4.31, all right-handed) remaining for analysis.
Non-participation	n/a
Randomization	n/a

Reporting for specific materials, systems and methods

We require information from authors about some types of materials, experimental systems and methods used in many studies. Here, indicate whether each material, system or method listed is relevant to your study. If you are not sure if a list item applies to your research, read the appropriate section before selecting a response.

Materials & experimental systems

n/a	Involved in the study	
☒	☐	Antibodies
☒	☐	Eukaryotic cell lines
☒	☐	Palaeontology and archaeology
☒	☐	Animals and other organisms
☒	☐	Clinical data
☒	☐	Dual use research of concern
☒	☐	Plants

Methods

n/a	Involved in the study	
☒	☐	ChIP-seq
☒	☐	Flow cytometry
☐	☒	MRI-based neuroimaging

Plants

Seed stocks	n/a
Novel plant genotypes	n/a
Authentication	n/a

Magnetic resonance imaging

Experimental design

Design type	Block-design

Design specifications

Each participant went through one resting-state run and three runs recorded during solving items from Raven's Standard Progressive Matrices (RSPM) as well as items from Raven's Advanced Progressive Matrices (RAPM)
Each RPM run included 10 items that were pseudo-randomly sampled from a set of 30 items that were identical for each participant. Each item was presented for a maximum time of 15 seconds without showing the solution possibilities. Participants were instructed to push a button once they had solved the item. After pushing the button or 15 seconds had passed, a solution set appeared on the screen. Participants were then required to select the solution they believed to be correct by pressing one of four buttons, corresponding to the index and middle fingers of either hand. Only one response was permitted. Between each item, there was a period of 12 to 15 seconds during which a fixation cross was shown.

Behavioral performance measures

RPM performance scores (percentage of correctly solved items)

Acquisition

Imaging type(s)

T2*-weighted functional images

Field strength

3-T

Sequence & imaging parameters

Functional MRI data were acquired using a 3T Siemens Trio scanner. Specifically, T2*-weighted functional images were obtained with a gradient-echo echo planar imaging sequence (echo time: 29 ms, repetition time: 2 s, 75° flip angle, 3.5 mm slice thickness, 30% distance factor, 240 mm field of view, voxel size: 3.8 mm × 3.8 mm × 3.5 mm). Resting-state runs lasted 5 minutes and 16 seconds, whereas RPM runs had a length of 5 minutes and 50 seconds.

Area of acquisition

Whole brain (cortical areas)

Diffusion MRI ☐ Used ☒ Not used

Preprocessing

Preprocessing software

FMRIPREP version 20.0.7

Normalization

Spatial normalization to the ICBM 152 Nonlinear Asymmetrical template version 2009c (RRID:SCR_008796; Fonov et al., 2009) was performed through nonlinear registration with the antsRegistration tool of ANTs v2.1.0 (RRID:SCR_004757; Avants et al., 2008), using brain-extracted versions of both T1w volume and template.

Normalization template

ICBM 152 Nonlinear Asymmetrical template version 2009c

Noise and artifact removal

Preprocessing steps comprised high-pass filtering at 0.008 Hz and a nuisance regression strategy including 24 head motion regressors, and 10 components (aCompCor, five white matter and five cerebral fluid) from a principal component analysis (PCA) to putative nuisance signals. For data recorded during the RPM, basis-set task regressors were applied simultaneously with the nuisance regressors to remove task-evoked neural activity.

Volume censoring

n/a

Statistical modeling & inference

Model type and settings

Relationship between RPM-specific centrality measures (participation coefficient and degree – based on functional connectivity) and RPM performance. Centrality measures were computed for 200 cortical nodes (200 node Schaefer parcellation).

Effect(s) tested

Partial Spearman correlations (controlled for age sex, head motion) between centrality measures and RPM performance for each of the 200 nodes.

Specify type of analysis: ☒ Whole brain ☐ ROI-based ☐ Both

Statistic type for inference

(See Eklund et al. 2016)

n/a

Correction

FDR

Models & analysis

n/a	Involved in the study
☐	☒ Functional and/or effective connectivity
☐	☒ Graph analysis
☒	☐ Multivariate modeling or predictive analysis

Functional and/or effective connectivity

Functional connectivity between all 200 cortical nodes was estimated as Fisher-z-transformed Pearson

Functional and/or effective connectivity	correlation between z-standardized BOLD signal time series of a) the resting-state run, and b) the three concatenated RPM runs (including both correct and incorrect trials).
Graph analysis	Two graph-theoretical centrality measures, degree and participation coefficient, were calculated for each node using the MATLAB-based Brain Connectivity Toolbox (BCT) to capture a) the overall connectedness of a brain region within the network and b) this region’s involvement in connections linking different brain systems (diversity of intermodular connectedness). Both measures were determined on proportionally thresholded (50%) functional connectivity matrices. For testing whether the threshold critically affects results, all analyses were repeated with proportional thresholds of 40% and 60%.
